# Unravelling the factors decisive to the implementation of EPODE-derived community approaches targeting childhood obesity: a longitudinal, multiple case study

**DOI:** 10.1186/s12966-016-0423-5

**Published:** 2016-09-05

**Authors:** Maria Rianne van der Kleij, Mathilde Crone, Ria Reis, Theo Paulussen

**Affiliations:** 1 Department of Public Health and Primary Care, Leiden University Medical Center, Postbus 9600 zone V-0-P, Leiden, 2300 RC The Netherlands; 2 Academic Workplace (AWP) Public Health Zuid-Holland Noord, Leiden, The Netherlands; 3 TNO Innovation for Life, Expertise Centre Child Health, Leiden, The Netherlands; 4 The Children’s Institute, School of Child and Adolescent Health, University of Cape Town, Cape Town, South Africa; 5 Amsterdam Institute for Social Science Research, University of Amsterdam, Amsterdam, The Netherlands

**Keywords:** Children, Obesity prevention, Community approach, Implementation, Intersectoral collaboration, Process evaluation

## Abstract

**Background:**

Implementation of intersectoral community approaches often fails due to a translational gap between the approach as intended and the approach as implemented in practice. Knowledge about the implementation determinants of such approaches is needed to facilitate future implementation processes.

**Methods:**

The implementation of five EPODE-derived intersectoral community approaches was studied longitudinally. Semi-structured interviews were held with 189 community stakeholders from four sectors to elucidate which determinants influenced implementation, and if an to which extent determinants differed across communities, sectors and over time. A framework approach was used to analyze our data.

**Results:**

Twenty-two key determinants of implementation were identified. Facilitators named were mostly proximal (stakeholder level), and barriers were mostly distal (context level). Key determinants varied greatly across sectors and over time, especially between the educational & health care sector and the private, welfare & sports sector. Only ‘perceived importance of IACO goals’ was identified as an universal implementation facilitator.

**Conclusions:**

Striking differences in determinants were found across sectors and over time. Also, stakeholders expressed that possibilities to adapt the approach to the local context were needed to improve implementation. We therefore propose to develop sector- and time specific leads for implementation, which should be approved and amended (over time) by stakeholders. This so-called ‘mutual adaptation’ allows for the use of both scientific insights and practice-based knowledge, enabling program management and community stakeholders to collaboratively improve their implementation efforts.

## Background

To address the pressing issue of childhood obesity [1–3], the French ‘Ensemble Prevenons l’Obesité Des Enfants’ (EPODE) program was developed [4, 5]. The EPODE program is an Intersectoral community Approach towards Childhood Obesity (IACO). Its main objective is to address obesity determinants on the micro- (child), meso- (family) and macro level (community context), thereby accounting for the multi-factorial etiology of childhood obesity. EPODE also engages stakeholders from several sectors within the community to integrate its four major pillars; (1) social marketing, (2) establishment of public-private partnerships, (3) acquisition of political commitment and (4) guidance of the approach via a scientific evaluation [4]. EPODEs program methodology is described in more detail elsewhere [4, 5]. The EPODE approach appeared successful in reducing childhood obesity in two French pilot towns [6]. After this success, the approach was scaled-up and various EPODE-derived approaches were launched worldwide [5]. The Dutch developed the EPODE-derived JOGG approach (an acronym for Youth On a Health Weight, in Dutch), and as of yet 83 communities in the Netherlands have adopted this approach [7].

Although the implementation of the initial EPODE program led to promising results, similar IACOs have shown significantly less impact on health-related outcomes [8, 9]. This lack of impact could be due to a translational gap often reported between the program as intended and the program as implemented in practice, especially in case of complex community-based programs [10–13]. Translation of programs into practice generally follow a four-stage diffusion process, often referred to as ‘diffusion of innovations’ [14]. The first stage consists of ‘dissemination’; actively promoting knowledge-awareness about a program among the target population. This stage is followed by ‘adoption’; in which the stakeholder decides whether or not to accept and use the program. During ‘implementation’, the program is put into use. The final stage, ‘continuation’ concerns the extent to which initial program implementation is continued. This process of diffusion is dynamic and users go through stages iteratively. A user can for example halt program implementation, but later decide to re-adopt and restart implementation. This study focusses specifically on the stages of implementation and continuation. We will refer to these stages combined as ‘the implementation process’.

To gain insight into the implementation process of IACOs, a pragmatic process evaluation is warranted. A process evaluation can help elucidate which determinants influence the implementation process [11, 13, 15, 16]. As of yet, a variety of determinants affecting the process of implementation of health promotion programs in general have been identified [17, 18]. For instance Fleuren et al. [19] constructed a framework that clusters 50 determinants of implementation of public health innovations. These determinants are split into four categories; the characteristics of the (1) adopting person (user), (2) innovation, (3) organization and (4) socio-political context.

Although some knowledge has been developed on the implementation of public health innovations in general, research on the implementation process of IACOs is still in its infancy. Only a limited number of studies have evaluated IACO implementation, and those that did have mostly focused on a single case, were performed at one moment over time, and assessed determinants of the implementation process in only one or two sectors [20].

To gain more insight into the process of IACO implementation, we studied the determinants of implementation of five EPODE-derived IACOs in the Netherlands. We evaluated whether and to which extent these determinants differed between communities, sectors and over time.

## Methods

The design of our research was guided by the framework of Saunders et al. [21]. This framework allows for an iterative adjustment of methods in accordance with local developments and the results of preliminary data analysis.

### Setting

**Fig. 1:**
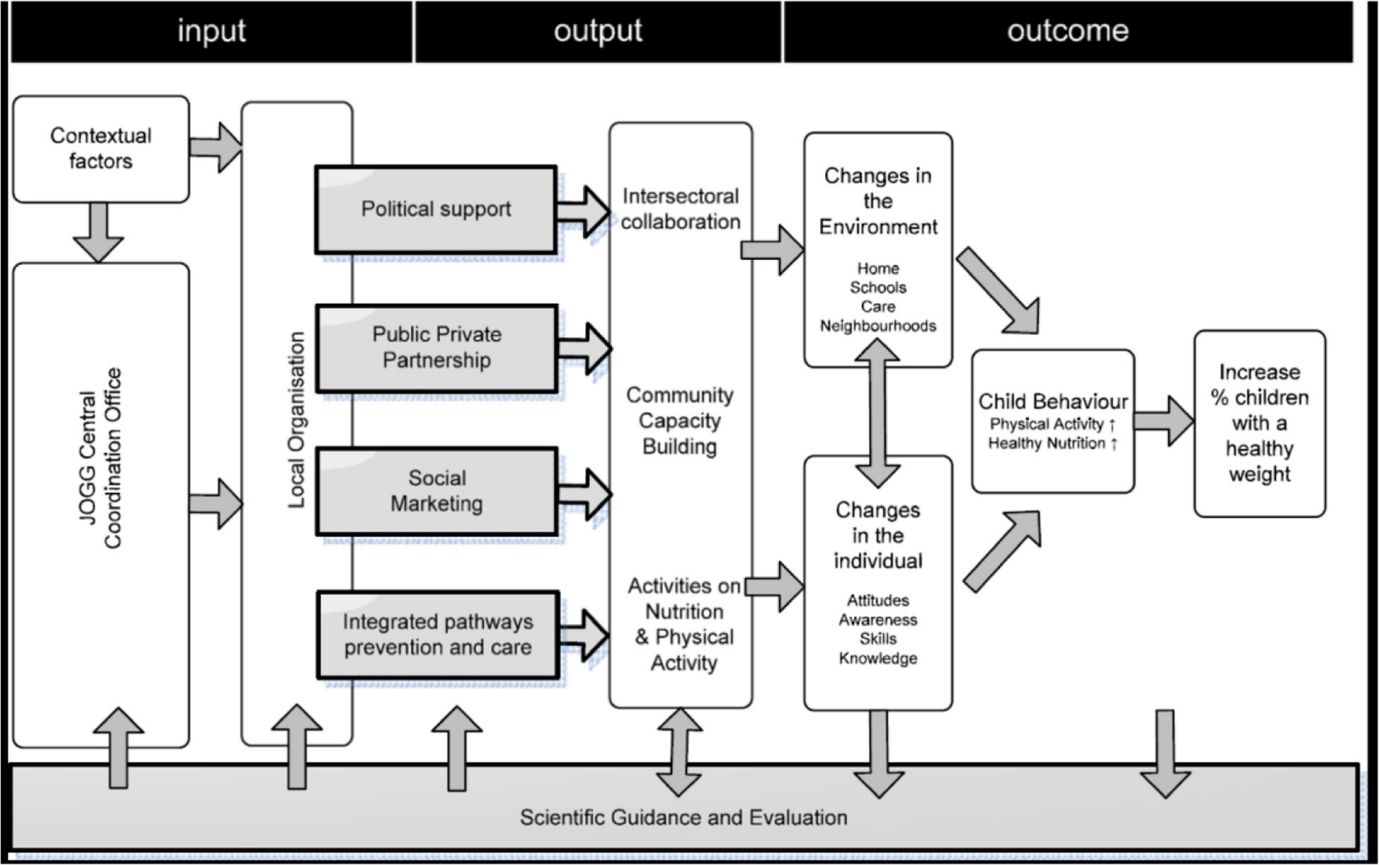
EPODE pillars & program methodology, van Koperen et al. [4]

Five communities implementing EPODE-derived IACOs were included in this study. Following principles of purposeful sampling [22], inclusion of communities was based on opportunity, willingness to participate and creating on diversity in our sample. Three of the included communities were implementing an IACO based on the JOGG approach, whereas the two other communities implemented an EPODE-derived IACO not commissioned by the national JOGG project office. Moreover, the IACO implemented within community I targeted merely the promotion of healthy nutrition, whereas the IACOs implemented in communities II to V targeted both physical activity and nutrition. The extent to which the IACOs were protocolled also differed. The IACO implemented in community I was partly protocolled. Hence, instructions were provided on ‘what’ to do (EPODE pillars, Fig. 1) and also partly on ‘how’ to deliver activities. IACOs within communities II to IV were not protocolled; The program manager only informed stakeholders on ‘what’ goals needed to be accomplished, but not on ‘how’ to accomplish them. Stakeholders were instead asked to integrate the EPODE pillars in existing activities, or to establish new activities that served the EPODE goals. Furthermore, the target population differed across the included IACOs; I and III targeted children 0–12 years of age, whereas II, IV and V targeted children between 0 and 18 or 19 years of age. Finally, the degree of involvement per sector varied. For example more than five stakeholders from the educational sector were actively implementing IACO activities within communities I, IV and V, whereas only one stakeholder from the education field was actively implementing IACO activities in community III (Table 1).

**Table 1:** Characteristics included communities & IACOs

Community	I	II	III	IV	V
Type of IACO				IACO based on	IACO based on
Implementation site	Neighborhood	Neighborhood	Neighborhood	Municipality	Municipality
Target population	0–12 years	0–19 years	0–12 years	0–18 years	0–18 years
Focus	N	PA & N	PA & N	PA & N	PA & N
Setup IACO	Partly protocolled	Not protocolled	Not protocolled	Not protocolled	Not protocolled
# inhabitants implementation site	27.400	13.325	7.345	18.216	40.958
Interventions that were included in our study per sector					
Educational	Fruit & water campaign	Preschool gardening & healthy N program	Integrated, multidisciplinary program elementary schools *(Nicely Fit!)*	Integrated, multidisciplinary program elementary schools *(Score for Health)* (pre)school PA & N policies	Integrated, multidisciplinary program elementary schools *(Score for Health)*
Health Care	Fruit & water campaign	Healthy N resilience program	Children’s physical therapy ‘toddler gym’	Children’s physical therapy ‘toddler gym’	-
Welfare & sports	Fruit & water campaign	Municipal PA & N ‘stimulation & connecting’ program	Integrated ‘active communities’ PA program Free running	Afterschool PA intervention N activities	Walk & run together community PA program ‘Try a sport you like’ community PA program
Private	Fruit & water campaign	Sponsoring of PA & N activities	Weight watchers class teen moms ‘Soup-making’ healthy N activity Sponsoring of PA & N activities (e.g. funding school playground)	School supermarket visits Football club initiated PA activities	School supermarket visits Football club initiated PA activities

*PA* physical activity, *N* nutrition

### Design

The implementation process of the included IACO’s was prospectively studied during several 6-monthly research waves. Five research waves were held in community A, four in community B, three in community C, and two in communities D & E. The number of research waves varied because the starting point of implementation differed across communities, while all research activities needed to be completed within the time frame of the research project. As IACOs are dynamic and always in transition, stakeholders included during the first research waves were not always ‘initial implementers’ (having less than 12 months of experience), and those included third, fourth or fifth wave were not always ‘continuing implementers’ (having more than 12 months of IACO implementation experience). To counter this issue and facilitate analysis, we therefore asked every participant how much experience they had with the implementation of that particular activity, and divided them into those having 12 or less months of experience with implementing the IACO (initial implementation), versus those having more than 12 months of IACO implementation experience (continued implementation).

After inclusion the research started with a baseline assessment of the IACO. We formulated a ‘state of affairs document’ including a description of IACO objectives, a list of participating community stakeholders and a list of the planned IACO activities. This document served as input for tailoring research methods and instruments to the local context. After this assessment, research waves were performed every 6 months. Every wave consisted of semi-structured interviews with stakeholders. Interview were based on a topic-list derived from the framework of Fleuren et al. [19]. At the start of each research wave, alterations or additions to this topic list were made based on the outcomes of the preceding wave. Verbal informed consent was obtained prior to the start of each interview and audio-recorded. We chose to obtain verbal consent instead of written consent as it is generally acceptable if no significant risks are involved for participants [23], and because it allowed for the (early) establishment of a bond of trust between researcher and participant. Moreover, it provided opportunity for participants to discuss any uncertainties or lack of clarity. All interviews were held face-to-face and were audio recorded, and duration varied from 15to 90 min, depending on the time available per stakeholder.

### Sample

Stakeholders were invited to participate in our study if they implemented at least one IACO activity that met all of the following criteria:

1. The activity was part of the IACO (according to the project manager) or was financed by IACO management,
2. The activity took place within the community boundaries,
3. The activity comprised direct contact with the target population.

Due to limited resources and finances, not all stakeholders meeting the inclusion criteria could be invited. Priority for inclusion was therefore assigned to those stakeholders that implemented IACO activities expected to be most important to reach the intended health outcomes. For example reach of the activity, evidence available for the efficacy of the activity, and whether the IACO activity was recurrent were taken into account. Stakeholders were invited to participate either via telephone or email. Fourteen stakeholders declined participation. Reasons for non-participation were mostly related to a lack of time or research fatigue [24]. A total of 189 stakeholders were included in our study: 89 (47 %) were embedded in the educational sector, 65 (34%) in the welfare & sports sector, 25 (13%) in the health care sector and 19 (10%) in the private sector. This sample mirrored the involvement of the different sectors within the IACOs at that time. In our sample, 82 (43%) stakeholders were implementing an IACO within community I, 27 (14.3%) in community II, 34 (18%) in community III, 28 (15%) in community IV and 18 (10%) in community V.

### Analysis

**Fig. 2:**
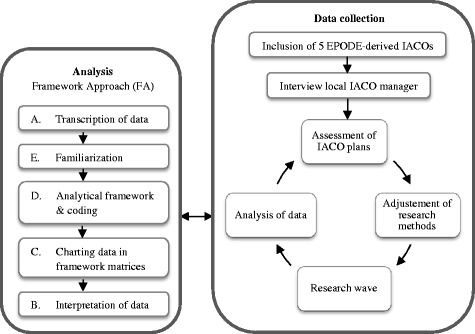
Research design

A four-stage Framework Approach (FA) [25] was used to guide our qualitative analysis (Fig. 2).

### Stage A: transcription

All audio-taped interviews were anonymized and transcribed verbatim. Anonymity of the participant was ensured by replacing the name of the participant with a number, and by not transcribing the names of other persons that were mentioned during the interview.

### Stage B: familiarization

All interviews were read in full text by two researchers (RK, SA) independently. Notes were made in the interview margin if any important segments were identified.

### Stage C: development of an analytical framework & coding

Atlas.ti for Windows version 6.2 (Scientific Software development, Berlin) was used to analyze our data. Coding was performed by two researchers and was primarily deductive; the framework by Fleuren et al. [19] was used to develop a code tree. Determinants emerging from our data that fell outside of the coding tree were added to the tree inductively. A document containing an operationalization of all codes was created and sequentially updated if new codes emerged.

The process of analysis commenced with the coding of one transcript by the two researchers jointly, to ensure both researchers interpreted and used codes in an uniform manner. The further process of coding was performed by the two researchers separately. Any discrepancies in coding were debated in person until consensus was reached.

### Stage D: charting the data into a framework matrix

Within- and cross-case displays as proposed by Miles & Huberman [26] were used to chart our data. A within-case display was formulated per interview (participant), and consisted of a short narrative followed by a description of the key barriers and facilitators. A determinant was considered a key barrier or facilitator if it became evident from the interview context that the stakeholder felt more strongly about a certain barrier or facilitator then other determinants. If for example a stakeholder stated that *‘lack of time was (one of) the most important thing that held me back from carrying out activity x’*, time was considered a key barrier. Derived from the individual within-case displays, cross-case displays per community, sector (sector categorization, additional information 1) and time period (initial and continued implementation) were established.

### Stage E: interpretation of the data

Cross-case displays were studied to (a) identify the most frequently named key barriers and facilitators per community and sector across time periods.

## Results

**Fig. 3:**
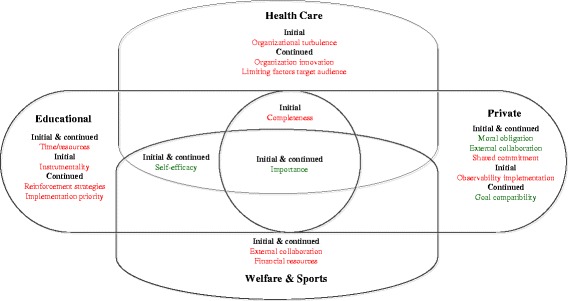
Visual display of unique and universal determinants across sectors

Twenty-two unique key determinants of IACO implementation were identified across communities, sectors and time periods. Thirteen key determinants were related to characteristics of the innovation and user, whereas nine determinants were related to the innovations strategy, organization and community & context. Facilitators were mostly user-related, barriers were for the greater part related to innovation (strategies), organization and context. An overview of all key determinants, their operationalization and illustrative quotes is provided in Table 2. Key determinants identified per time period and sector are also displayed in Table 2. Figure 3 illustrates similarities and differences in key determinants per sector and over time.

**Table 2:** Overview of key determinants per sector, community and in time

Direction	Category	Determinant	Operationalization	Comm	Initial implementation ^a^				Continued implementation ^a^			
					Edu	HC	WS	Priv	Edu	HC	WS	Priv
					*n* = 43	*n* = 9	*n* = 33	*n* = 8	*n* = 37	*n* = 14	*n* = 32	*n* = 11
Facilitator	User	Importance	Feeling that IACO goals are of importance	I–V	61	55	79	100	70	86	53	73
	User	Self-efficacy	Beliefs about the ability to reach IACO goals	I–V	47	33	36		41	36	40	
	Innovation	Uptake into routine	Possibility to integrate IACO activities into daily working routine	I, II, V			30		38	21		
	Innovation	Possibilities to adapt	*Possibility to adapt non-essential elements of IACO*	I–IV	54						22	
	User	Moral obligation	Having considerations, stemming from personal values, about whether it is ‘right’ or ‘wrong’ to implement the IACO	I–III				68				
	User	Goal compatibility	*Compatibility of IACO goals with organizational or user goals*	I–V								46
	Community & context	External collaboration	*Collaboration community stakeholders with respect to IACO*	I–V				38				
	Innovation	Compatibility	Level to which IACO activities are compatible with pre-existing practices	I–IV		44						18
Barrier	Innovation	Completeness	Completeness of IACO activities (e.g. parent meeting) and materials (i.e. manuals, gadgets)	I–IV	35	44		50			22	
	Community & context	Shared commitment	*Feeling of shared commitment with community partners for IACO implementation*	I–V				38				46
	Innovation	Procedural clarity	Level in which IACO procedures are clear	I–V			30					46
	Community & context	(Anticipated) response target population	Level of participation of children and parents in IACO activities	I–V						28		36
	User	Time/resources	Availability of time/resources to implement IACO	I–V	28				27			
	Community & context	External collaboration	*Collaboration community stakeholders with respect to IACO*	I–V			27				25	
	Organization	Financial resources	Availability financial resources organization to implement IACO	II–V			27				22	
	Community & context	Observability implementation	*Observability of IACO implementation by other community stakeholders*	III				68				
	Innovation	Management innovation	Management/organization of innovation	I, III, V						43		
	Innovation strategies	Reinforcement strategies	Reinforcement strategies to promote ongoing IACO use (e.g. a training or new promotional materials)	I–V					36			
	Organization	Organizational turbulence	Changes in organization affecting IACO implementation (e.g. reorganization, cuts)	I–V		33						
	Innovation	Compatibility	Level to which IACO activities are compatible with pre-existing practices	I–V		33						
	Innovation	Instrumentality	Quality and durability of materials	I, IV	30							
	User	Implementation priority	Priority assigned to implementation of IACO	I–IV					30			
	Community & context	Limiting factors target population	*Level to which limiting factors (*i.e. *behavioural, financial problems) are present in target population*	I, III, V						29		

*Comm* Communities, *Edu* Educational sector, *HC* Health Care sector, *WS* Welfare & Sports sector, *Priv* Private sector

^a^ Percentage of stakeholders naming the key determinant is displayed

### Similarities in determinants across sectors, communities and over time

High perceived importance of IACO goals was identified as a key facilitator across all communities, sectors and over time.

No, it (the IACO) is really a passion of mine, a personal motive, we just want to make the world a better place. Not on a large scale, just starting small. To help them (children) feel ‘I am worth it, and I have a good quality of life’. *(Youth welfare worker, community I)*

A high level of self-efficacy for IACO implementation was also identified as a key facilitator in all communities and over time periods, but not for the private sector.

You have to make a detour for it (the implementation) to work. You have to water your garden, attend to the plants and keep an eye on the children. So, it demands more of you, but it is worth it! *(Preschool teacher, community II)*

The following barriers were named to impede implementation in all five communities, but not in all sectors and time periods: Incompleteness of innovation materials (such as sports equipment or gadgets such as water cans or stickers), low procedural clarity, lack of time and/or resources, organizational turbulence, minimal participation of the target population and lack of feeling a shared commitment with partners for IACO implementation.

### Sector-specific determinants

#### Educational sector

A lack of time and resources was only named as a barrier for implementation by educational stakeholders. The barrier was named in all communities, during both initial and continued implementation.

Lately, management has been tinkering with our working hours, We have to undertake all sorts of activities we absolutely don’t have time for. And this (the IACO) is then typically something that doesn’t get done. If we would just get two hours or so to prepare for it, but that’s just not going to happen. If we would get extra hours, then I think implementation of the IACO would be compatible with our regular program. *(Teacher, community V)*

Educational sector stakeholders explained that they were primarily held accountable for the academic performance of their students, and not the prevention of health-related risks

We have been so busy the last couple of years, at a certain moment you think ‘I don’t even know the name of this student in my class. So I think.. Yes, our main priorities lie elsewhere, not with this water campaign. *(Teacher, community I)*

Low quality and durability of the IACO materials (instrumentality) was only mentioned to impede implementation by educational stakeholders. Also, solely educational stakeholders expressed during continued implementation that a low priority assigned to IACO implementation (communities I–IV) and a lack of reinforcement strategies (all communities) were key barriers. Educational stakeholders mentioned they needed a continuous reinforcement of IACO implementation because of the low priority they assigned to implementation and their perceived lack of resources.

The project has been put on the back burner. We need someone who will say to us: Do you still focus on the implementation of the project? *(Teacher, community I)*

A facilitator for educational stakeholders during initial implementation was the possibility to adapt non-essential elements of the intervention.

One of my colleagues has bought an extra elastic band, a large band. This band prevents that the cover of our school garden gets blown away by the wind; the provided tie-wraps just didn’t work for us. (Preschool teacher, community II).

#### Health care sector

Health care stakeholders mentioned that ‘organizational turbulence’ caused by the recent merge of the majority of their local youth health care facilities, impeded the implementation process of the IACO.

But you know, because of the relocation, everything (campaign materials) has been stored into cabinets. And nobody has really finished unpacking. So, it is just a bit like: I accidently found another campaign bag, let’s give that to the next patient. *(Youth health nurse, community I)*

Perceived behavioral or financial problems of the target population were identified as key barriers for implementation by the stakeholders in health care, in communities I, III and IV.

It’s the general attitude of parents, they are difficult to reach. Some parents just don’t want to change. They don’t go to any health care provider. They just think: it (obesity) will pass. That does not facilitate my implementation of the intervention. *(Children’s physical therapist, community IV)*

Formal uptake of IACO activities into their daily working routine was also solely mentioned as an implementation facilitator by health care stakeholders, in three out of five communities.

#### Welfare & sports sector

Unsound collaboration, for instance a perceived lack of response from other stakeholders when collaboration was initiated, was identified as a key barrier to initial implementation only for stakeholders embedded in the welfare and sport sector. Inadequate financial resources was also only identified as a key barrier to these stakeholders.

We just perform our own activities, and that’s it. I do think we could make a lot of progress (with IACO implementation) if we would work together as community partners. I really believe that would make a difference! *(Youth welfare worker, community III)*

Stakeholders from this sector also stated that implementation was sometimes hindered by a lack of procedural clarity, for example caused by insufficient information being available on when and how certain IACO activities needed to be performed.

What is exactly the goal of JOGG, what do they want to achieve? And in which manner? This remains totally unclear to me. *(Welfare worker, community II)*

#### Private sector

Exclusively for private sector stakeholders, solid collaboration with community stakeholders was named to facilitate implementation. Solid collaboration to them meant for instance ‘*smooth communication between stakeholders on the division of tasks’* and ‘*the reciprocal sharing of resources or facilities with other stakeholders’*. Feeling morally obliged to implement the IACO was also identified as a key facilitator, during both continued and initial implementation.

I own a commercial enterprise, but I also find it important that children are healthy. Therefore, I will provide the (health promotion) training to teenage mothers almost for free. It is just necessary to be a socially responsible entrepreneur. *(Owner private enterprise, community III)*

Only within this sector, continued implementation was facilitated by a high compatibility of stakeholders’ and IACO goals. Stakeholders for example stated that by providing fruit free of charge (IACO goal), they expected to make more profit as parents would be enticed to buy other products in their store (own goal). Finally, invisibility of the IACO implementation of other stakeholders and feeling no shared commitment for IACO implementation with these stakeholders were identified as private-sector specific key barriers.

I think that is of importance to, well to get a clear story. To gather all community partners every couple of months and discuss ‘what are we going to do?’ or ‘what vision do we want to project? Because at the moment, I have no clue about what happens in the schools or at the Centre for Youth & Family. *(Supermarket manager, community I)*

## Discussion

This study aimed to elucidate which determinants are decisive for IACO implementation, and if differences across communities, sectors and over time were present.

Twenty-two key determinants of IACO implementation were identified; 13 barriers, seven facilitators and two were identified as both a facilitator and barrier. Facilitators were mostly internal (stakeholder level), whereas barriers were mostly external (innovation context). Key determinants varied to a great extent across sectors and over time. Striking differences in sector specific determinants were found; Determinants named by stakeholders embedded in the private and welfare & sports sector were most often related to the context and organization level, whereas educational and health-sector stakeholders attributed barriers more often to the intrinsic characteristics of the innovation. Only one determinant, perceived importance of attaining the IACO’s goals, was identified as a facilitator across all sectors, communities and time periods.

### Interpretation of findings

This study showed that IACO implementation determinants were in large part sector and time specific. This is a new finding, as previous studies have mostly focused on IACO implementation in general and not on implementation within specific sectors and over time [20].

Specifically for the private, welfare- and sports sector, determinants related to the ‘community and context’ were found to influence IACO implementation. For instance, (un)sound collaboration with community partners was only named by these stakeholders as a key determinant, and not by educational- and health care stakeholders. This could reflect the nature of the IACO activities prescribed; for example the football club embedded in community III needed to collaborate intensively with the local welfare organization to recruit participants and to ensure the use of certain facilities. In contrast, education and health care stakeholders were prescribed IACO activities that required little collaboration and that could mostly be performed within their own setting. This indicates that collaboration is only perceived as a determinant to IACO implementation if participating stakeholders are dependent on other stakeholders for the set-up of their activities, resources, or the recruitment of participants. This is important to conclude, as IACO implementation is then partly dependent on the willingness of another stakeholder to collaborate or assist with the other stakeholders’ IACO implementation.

Private sector stakeholders stated that collaboration with community partners was a key facilitator to implementation, whereas welfare- and sports stakeholders perceived this as a key barrier. This difference could be based in the welfare- and sport stakeholders’ perception that the large effort needed to establish collaboration did not balance the anticipated target group benefits of implementation (‘outcome expectations [19]’). In contrast, private sector stakeholders viewed collaboration as a ‘significant effort’ but stated that this effort was balanced by the expected external (material) rewards (perceived external instrumentality [27, 28]). These external rewards emanating from collaboration were for example the extension of their clientele, and opportunity to meet new business partners. Moreover, solely private sector stakeholders named that a limited shared commitment with community stakeholders for and low visibility of their IACO implementation decreased their implementation efforts. Both of these determinants can be viewed as requirements to reach their perceived internal and external rewards linked to collaboration. For instance stakeholders might have expected that a lack of shared commitment would decrease their opportunities to extent their business network, and that a low visibility of implementation would reduce the opportunity to communicate ‘a positive company image’ to potential clients. Also, only private sector stakeholders mentioned that IACO implementation was facilitated because they felt morally obligated to implement the IACO. Subsequent feelings of being a ‘socially responsible’ entrepreneur could be considered as an internal reward. Striving to be a socially responsible entrepreneur and strengthening connections with potential business partners have also been named as most important motivators for private stakeholders’ IACO implementation by Leenaars et al. [29]. However, if and how perceived internal and external instrumentality generated by these determinants is the source of implementation motivation needs to be further explored.

Only for welfare- and sports stakeholders, a ‘lack of financial resources’ was identified as a key barrier to IACO implementation. Although the absence of financial resources is a widely cited barrier to implementation of IACOs [30–35], it has not previously been identified in specific for this sector. We argue that the availability of finances could be a key barrier especially for the this sector, as they, of all sectors, are most dependent on external (government-based) subsidies. When our study was conducted, the Netherlands was in the middle of a financial recession [36]. This recession gave rise to a significant decline in governmental and municipal financial support and subsidies, especially those that were not considered to facilitate basic needs (such as health care and education). This might explain why ‘a lack of finances’ was such a prominent barrier to implementation for welfare stakeholders. However, it should be noted that all organizations that rely on government based-subsidies, and not only welfare- and sports organizations, could be at risk for IACO implementation failure if subsidies are cut or withdrawn.

Attributes of the target population, for example the presence of financial or motivational problems, were only named as barriers by health care stakeholders. Other studies also reported on the influence of target population attributes on IACO implementation [34, 35, 37], but not with reference to a specific sector. Lack of motivation and compliance of patients is however frequently reported as an impeding factor for the integration of preventative activities in the daily practice of health care professionals [38–41]. Moreover, studies have shown that primary care providers often feel ill-equipped to improve the motivation of children and parents and are concerned that raising the issue might damage the patient-provider relationship [42]. Countering these attitudes and beliefs and thereby improving the self-efficacy of health care stakeholders has been demonstrated to facilitate implementation of childhood obesity counseling in primary care [43].

Educational stakeholders stated they had insufficient time and resources available to implement IACO activities, as they committed the limited time and resources available to ensure their students’ academic performance. Other studies also reported that demands teachers face with regards to students’ academic achievements can conflict with priority for health promotion in the school [44–48]. This priority dilemma also links to what is referred to as contextual integration in the Normalization Process Theory [49], meaning that the implementation and normalization of activities depends on how it relates to (the demands and context of) the organization it is implemented. Hence, although teachers might consider childhood obesity prevention as important, IACO-activities do not seem to agree with their primary task. Arguably related to the lack of priority, solely educational stakeholders expressed the need for continuous external reinforcement to sustain IACO-activities. This finding are in line with the results of a recent study from van Naussau et al. [48] on the implementation of the school-based obesity prevention approach DOiT. They found that the continued implementation of an obesity prevention approach in schools is influenced by opportunities to re-use intervention materials and incentives on how to continue implementation. Installment of an internal implementation coordinator who can manage and apply reinforcement strategies might be a solution to this problem [48]. This coordinator could then also function as the ‘first point of call’ for teachers who are in need of tips and tricks on how to implement activities when only limited time is available.

Across sectors, we found that IACO implementation was facilitated if a stakeholder perceives IACOs’ goals as important. This finding is corroborated by other studies examining IACO implementation [32, 34, 50–52]. However, less successful implementers also stated they felt the goals of the IACO were important. This might suggest that perceiving IACO goals as important is not a decisive factor to implementation, but that only in combination with other facilitators (or the absence of other barriers) implementation success can be achieved. High self-efficacy was also identified as a key facilitator to IACO implementation; across time and in three out of four sectors. Few previous studies have found this determinant to be of importance, only Davis et al. [53] mentioned self-efficacy influenced the implementation of the IACO ‘Head Start’. Self-efficacy is however empirically tested as a highly relevant determinant in many other innovation studies, outside the context of IACO’s, as it is accounted for by several implementation frameworks (such as the Fleuren framework [19] used in this study) and theories of behavior change [54, 55].

### Strengths & weaknesses

This study is the first to systematically [21] evaluate determinants of IACO implementation in multiple communities, sectors and over time. Moreover, in concordance with the latest insight on how to best prevent childhood obesity [16], this study gives a voice to a large sample of community stakeholders on what is important and feasible to them when it comes to IACO implementation.

Another strength of this study is the iterative adjustment of research methods, in line with local community developments. This allowed us to fine-tune our data collection, and to gain a more internally consistent evaluation of IACO implementation determinants.

Several strategies were adopted to generate optimal reliability and internal validity of our data [26, 56]. All interviews were recorded and transcribed verbatim, and data analysis was performed by two researchers via a framework approach [25] using analytical software. Data was reduced in multiple steps through the formulation of narratives and within- and cross case comparisons [26].

Selection of participants in this study can be considered both as a strength and limitation. We included five communities in this study, which differed in size, childhood obesity rates and other characteristics. From these communities, a relatively large sample of stakeholders from diverse sectors was included. We therefore argue that we obtained the most diverse and representative sample possible considering local resources and opportunities, but do feel that this purposeful sampling might have given rise to selection bias. For example stakeholders that declined participation often indicated they were experiencing research fatigue [24] or time limitations. One could then hypothesize that stakeholders who did agree to participate were more motivated or les strained by their workload. Also, because the community setting is dynamic, we were not able to follow the same participants over time. We for example encountered a high staff turnover in several organizations or a rapid change in policy causing a halt in IACO implementation. To counter these challenges, we compared stakeholders based on the time they were implementing the IACO and made a cross-sectional comparison of data.

Finally, we used a semi-action research design; we provided an overview of study results to community stakeholders following every research wave, without advocating if and what changes should be made to IACO implementation plans. This approach was chosen to empower community members as much as possible, whilst keeping data contamination minimal. Solely presenting the results to the stakeholders initiated some changes in implementation plans, but not all results could be translated into practice because stakeholders lacked the time and (human) resources to do so. We feel that, although this might lead to more data contamination, full Participatory Action Research (PAR) [57] would be a superior approach to use in future IACO implementation studies. Research and practice work together in PAR to translate research findings into implementation plans, enabling a swift transition of research finding into practice.

## Conclusions

The implementation of IACOs is both dynamic and complex. Different determinants influence IACO implementation over time and across communities and sectors. We therefore argue that a tailored implementation plan should be formulated per sector and in time, preferably using a ‘mutual adoption strategy [58]. Mutual adaption enables IACO program managers and community stakeholders to collaboratively improve implementation efforts, by combining both the latest scientific evidence and best practices. Moreover, stakeholders are asked to verify implementation plans during multiple points over time, ensuring an optimal fit with local needs and circumstances. This strategy has been reported to enhance the implementation of complex health promotion approaches in several other studies [59, 60]. Finally, we advise future research to use mixed methods and a participatory action research design to evaluate the use of tailored IACO implementation plans and to elucidate which implementation strategies best match these plans.

## Abbreviations

EPODE: Ensemble prevenons l’Obesité des enfants
IACO: Intersectoral community approach to childhood obesity
FA: Framework approach
